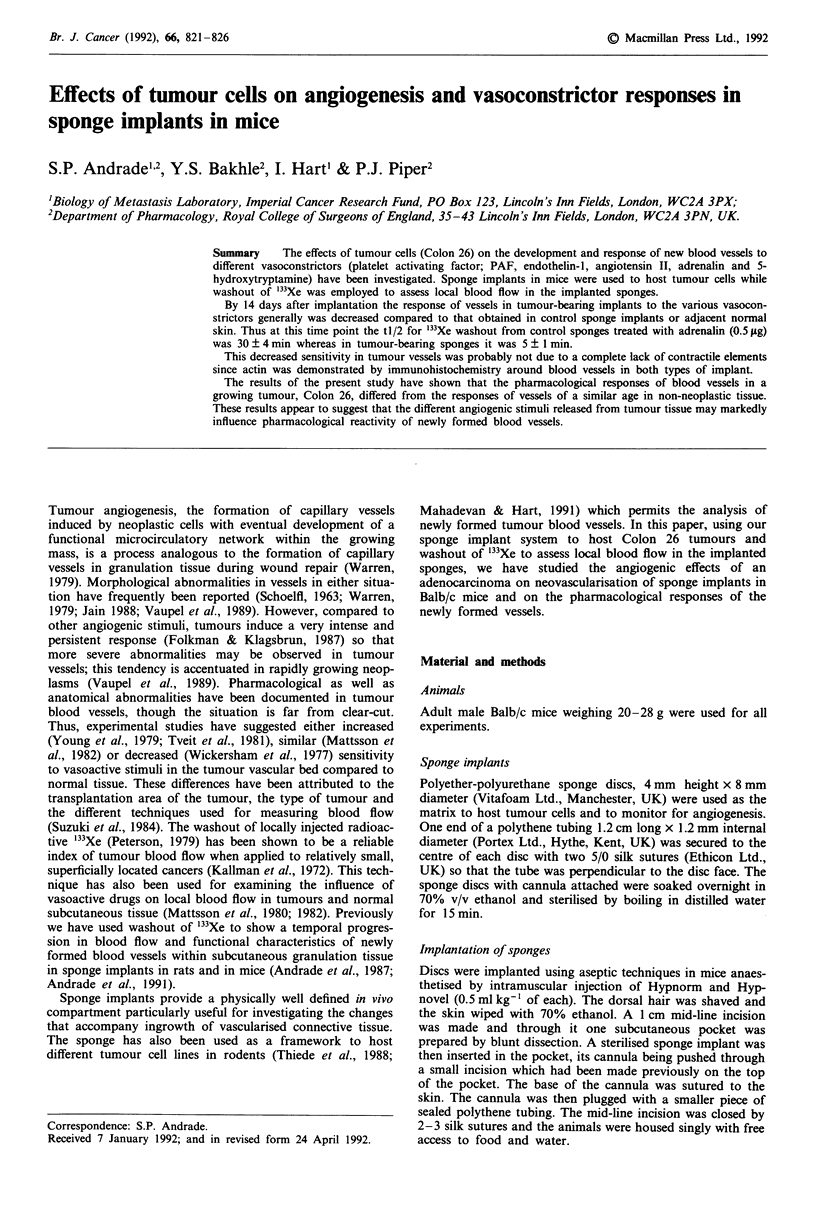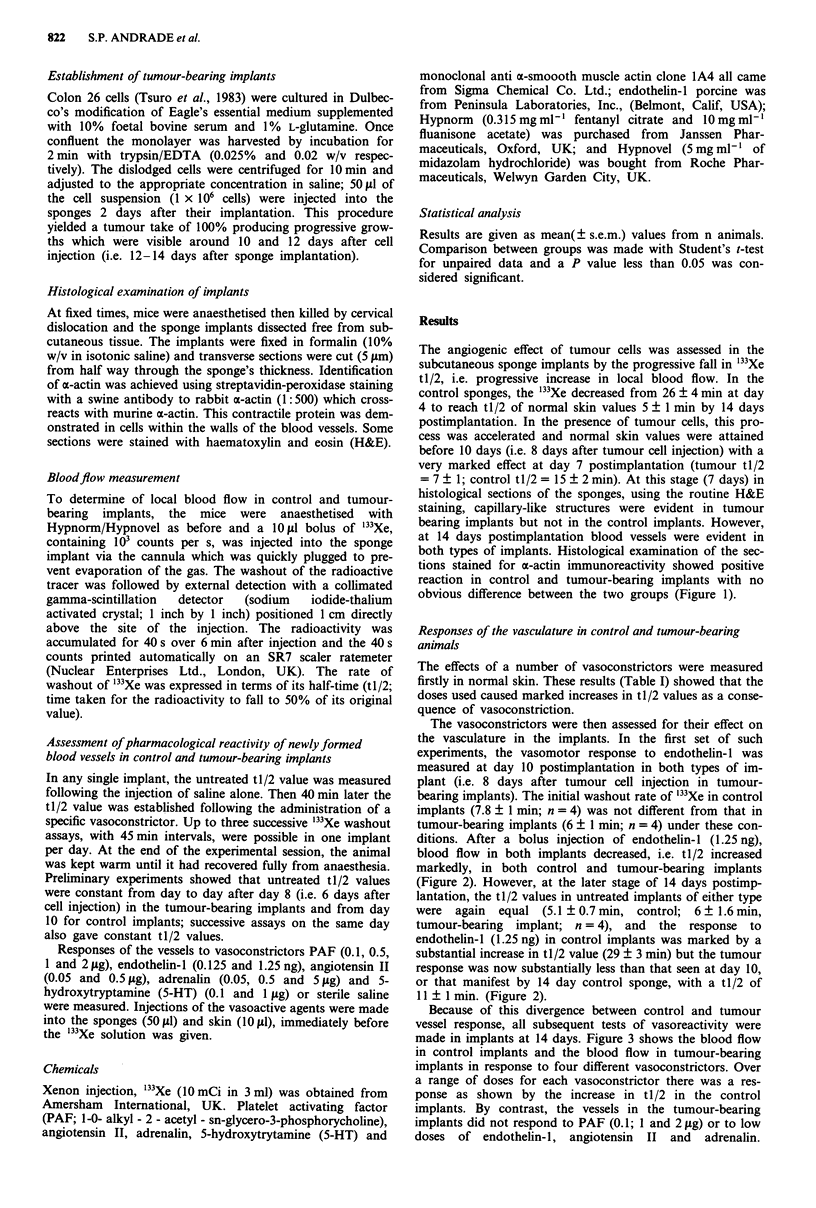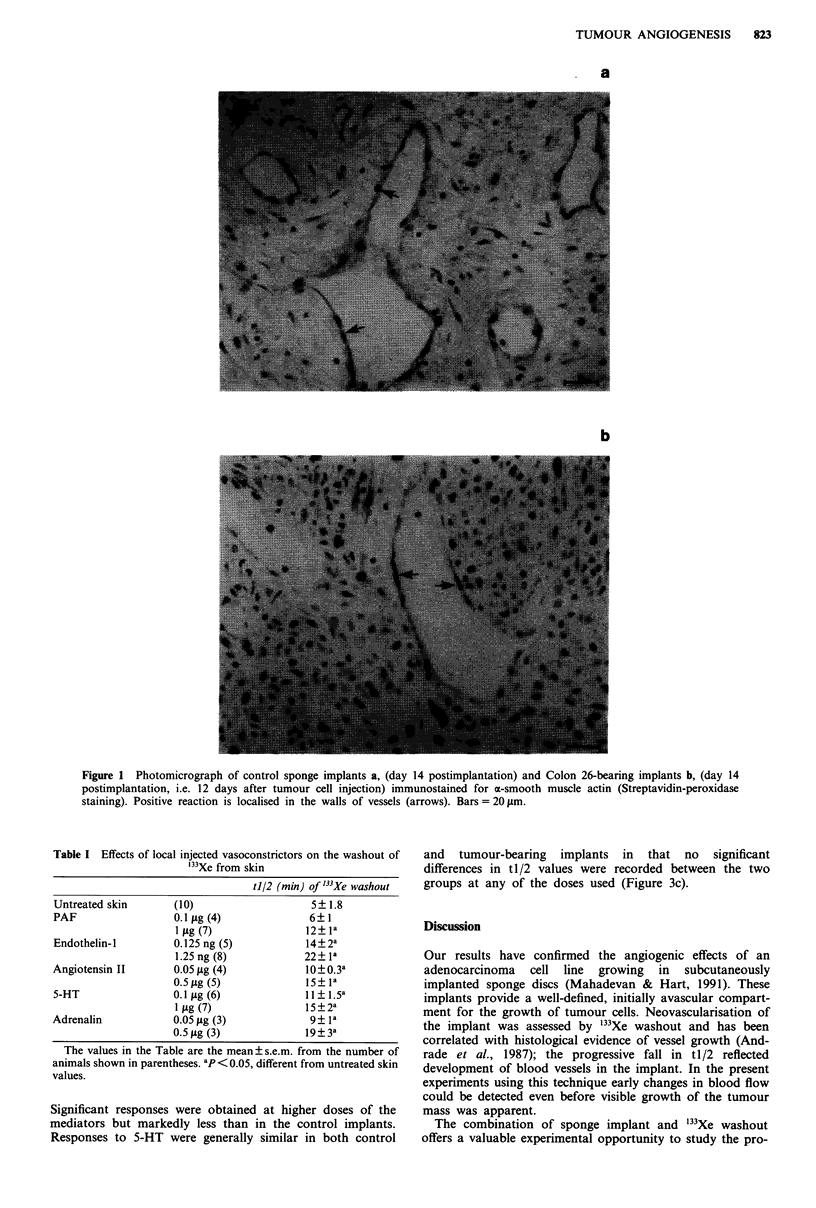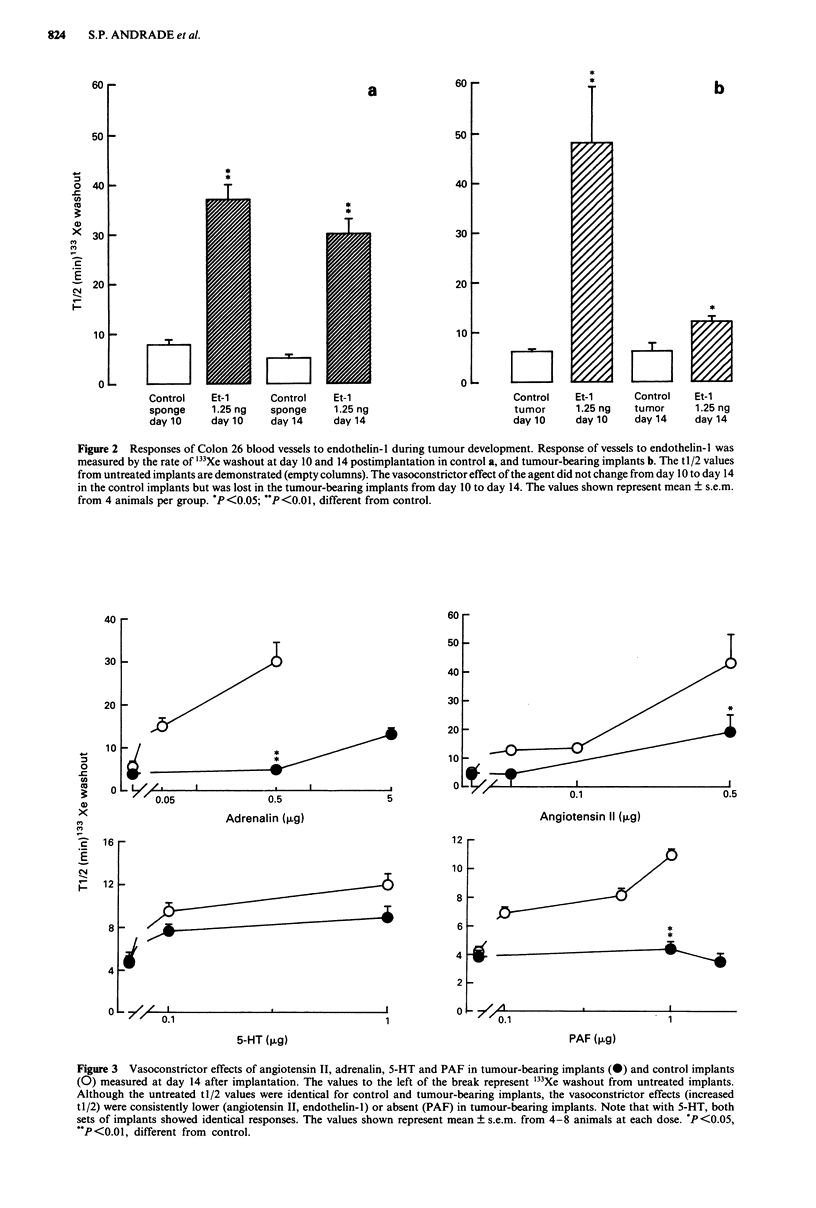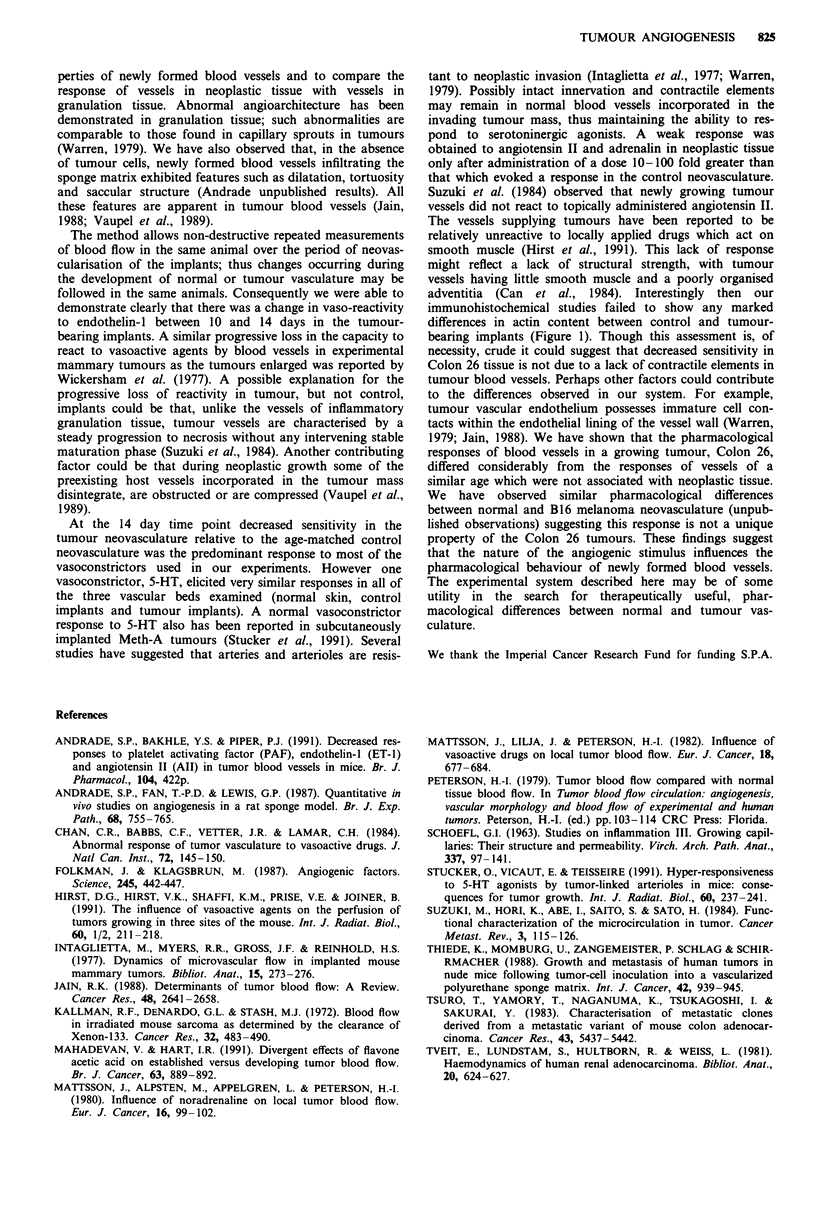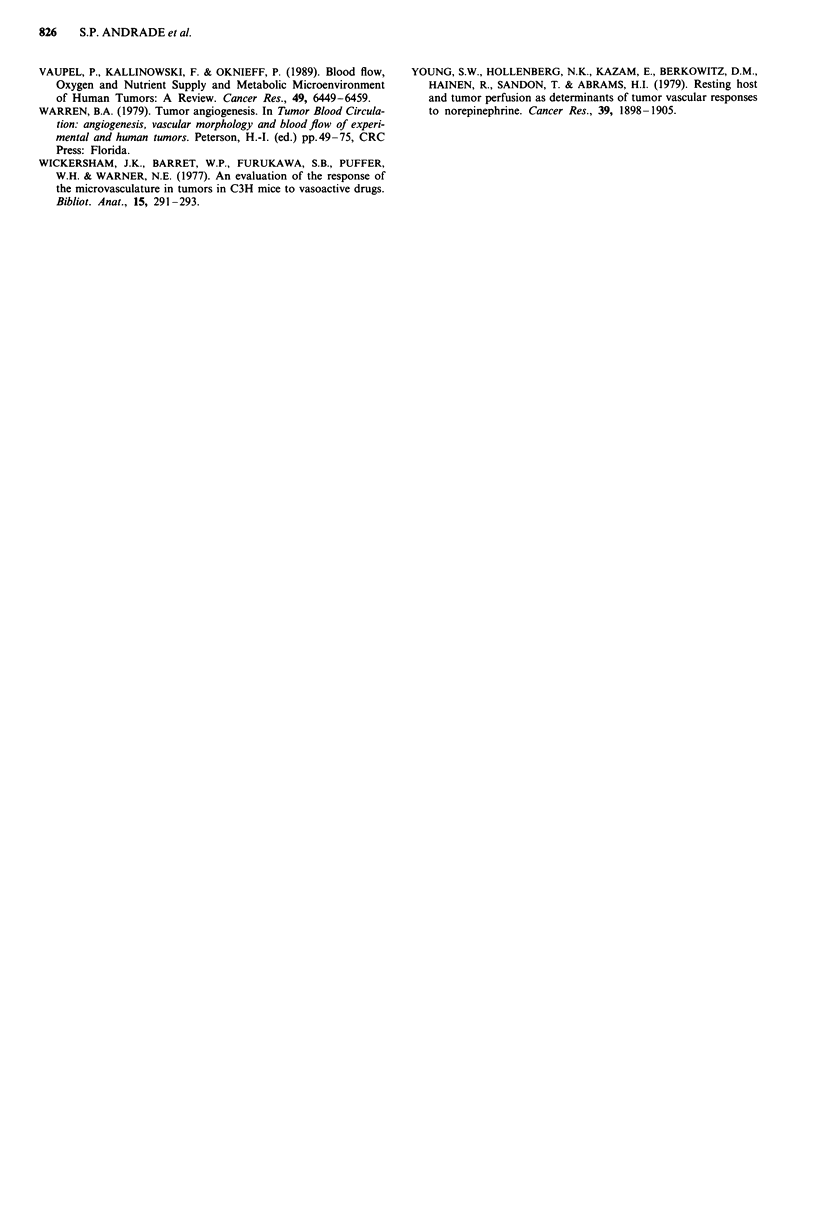# Effects of tumour cells on angiogenesis and vasoconstrictor responses in sponge implants in mice.

**DOI:** 10.1038/bjc.1992.367

**Published:** 1992-11

**Authors:** S. P. Andrade, Y. S. Bakhle, I. Hart, P. J. Piper

**Affiliations:** Biology of Metastasis Laboratory, Imperial Cancer Research Fund, London, UK.

## Abstract

**Images:**


### Effects of tumour cells on angiogenesis and vasoconstrictor responses in sponge implants in mice

S.P. Andrade[1,2], Y.S. Bakhle[2], I. Hart[1] & P.J. Piper[2]

[1]*Biology of Metastasis Laboratory, Imperial Cancer Research Fund, PO Box 123, Lincoln's Inn Fields, London, WC2A 3PX;* [2]*Department of Pharmacology, Royal College of Surgeons of England, 35-43 Lincoln's Inn Fields, London, WC2A 3PN, UK.*

**Summary** The effects of tumour cells (Colon 26) on the development and response of new blood vessels to different vasoconstrictors (platelet activating factor; PAF, endothelin-1, angiotensin II, adrenalin and 5-hydroxytryptamine) have been investigated. Sponge implants in mice were used to host tumour cells while washout of $^{133}Xe$ was employed to assess local blood flow in the implanted sponges.

By 14 days after implantation the response of vessels in tumour-bearing implants to the various vasoconstrictors generally was decreased compared to that obtained in control sponge implants or adjacent normal skin. Thus at this time point the t1/2 for $^{133}Xe$ washout from control sponges treated with adrenalin (0.5 µg) was 30 ± 4 min whereas in tumour-bearing sponges it was 5 ± 1 min.

This decreased sensitivity in tumour vessels was probably not due to a complete lack of contractile elements since actin was demonstrated by immunohistochemistry around blood vessels in both types of implant.

The results of the present study have shown that the pharmacological responses of blood vessels in a growing tumour, Colon 26, differed from the responses of vessels of a similar age in non-neoplastic tissue. These results appear to suggest that the different angiogenic stimuli released from tumour tissue may markedly influence pharmacological reactivity of newly formed blood vessels.

Tumour angiogenesis, the formation of capillary vessels induced by neoplastic cells with eventual development of a functional microcirculatory network within the growing mass, is a process analogous to the formation of capillary vessels in granulation tissue during wound repair (Warren, 1979). Morphological abnormalities in vessels in either situation have frequently been reported (Schoelfl, 1963; Warren, 1979; Jain 1988; Vaupel *et al.*, 1989). However, compared to other angiogenic stimuli, tumours induce a very intense and persistent response (Folkman & Klagsbrun, 1987) so that more severe abnormalities may be observed in tumour vessels; this tendency is accentuated in rapidly growing neoplasms (Vaupel *et al.*, 1989). Pharmacological as well as anatomical abnormalities have been documented in tumour blood vessels, though the situation is far from clear-cut. Thus, experimental studies have suggested either increased (Young *et al.*, 1979; Tveit *et al.*, 1981), similar (Mattsson *et al.*, 1982) or decreased (Wickersham *et al.*, 1977) sensitivity to vasoactive stimuli in the tumour vascular bed compared to normal tissue. These differences have been attributed to the transplantation area of the tumour, the type of tumour and the different techniques used for measuring blood flow (Suzuki *et al.*, 1984). The washout of locally injected radioactive $^{133}Xe$ (Peterson, 1979) has been shown to be a reliable index of tumour blood flow when applied to relatively small, superficially located cancers (Kallman *et al.*, 1972). This technique has also been used for examining the influence of vasoactive drugs on local blood flow in tumours and normal subcutaneous tissue (Mattsson *et al.*, 1980; 1982). Previously we have used washout of $^{133}Xe$ to show a temporal progression in blood flow and functional characteristics of newly formed blood vessels within subcutaneous granulation tissue in sponge implants in rats and in mice (Andrade *et al.*, 1987; Andrade *et al.*, 1991).

Sponge implants provide a physically well defined *in vivo* compartment particularly useful for investigating the changes that accompany ingrowth of vascularised connective tissue. The sponge has also been used as a framework to host different tumour cell lines in rodents (Thiede *et al.*, 1988; Mahadevan & Hart, 1991) which permits the analysis of newly formed tumour blood vessels. In this paper, using our sponge implant system to host Colon 26 tumours and washout of $^{133}Xe$ to assess local blood flow in the implanted sponges, we have studied the angiogenic effects of an adenocarcinoma on neovascularisation of sponge implants in Balb/c mice and on the pharmacological responses of the newly formed vessels.

### Material and methods

### *Animals*

Adult male Balb/c mice weighing 20-28 g were used for all experiments.

### *Sponge implants*

Polyether-polyurethane sponge discs, 4 mm height × 8 mm diameter (Vitafoam Ltd., Manchester, UK) were used as the matrix to host tumour cells and to monitor for angiogenesis. One end of a polythene tubing 1.2 cm long × 1.2 mm internal diameter (Portex Ltd., Hythe, Kent, UK) was secured to the centre of each disc with two 5/0 silk sutures (Ethicon Ltd., UK) so that the tube was perpendicular to the disc face. The sponge discs with cannula attached were soaked overnight in 70% v/v ethanol and sterilised by boiling in distilled water for 15 min.

### *Implantation of sponges*

Discs were implanted using aseptic techniques in mice anaesthetised by intramuscular injection of Hypnorm and Hypnovel (0.5 ml $kg^{-1}$ of each). The dorsal hair was shaved and the skin wiped with 70% ethanol. A 1 cm mid-line incision was made and through it one subcutaneous pocket was prepared by blunt dissection. A sterilised sponge implant was then inserted in the pocket, its cannula being pushed through a small incision which had been made previously on the top of the pocket. The base of the cannula was sutured to the skin. The cannula was then plugged with a smaller piece of sealed polythene tubing. The mid-line incision was closed by 2-3 silk sutures and the animals were housed singly with free access to food and water.

Correspondence: S.P. Andrade.
Received 7 January 1992; and in revised form 24 April 1992.

*Establishment of tumour-bearing implants*

Colon 26 cells (Tsuro *et al.*, 1983) were cultured in Dulbecco's modification of Eagle's essential medium supplemented with 10% foetal bovine serum and 1% L-glutamine. Once confluent the monolayer was harvested by incubation for 2 min with trypsin/EDTA (0.025% and 0.02 w/v respectively). The dislodged cells were centrifuged for 10 min and adjusted to the appropriate concentration in saline; 50 µl of the cell suspension ($1 \times 10^6$ cells) were injected into the sponges 2 days after their implantation. This procedure yielded a tumour take of 100% producing progressive growths which were visible around 10 and 12 days after cell injection (i.e. 12–14 days after sponge implantation).

*Histological examination of implants*

At fixed times, mice were anaesthetised then killed by cervical dislocation and the sponge implants dissected free from subcutaneous tissue. The implants were fixed in formalin (10% w/v in isotonic saline) and transverse sections were cut (5 µm) from half way through the sponge's thickness. Identification of α-actin was achieved using streptavidin-peroxidase staining with a swine antibody to rabbit α-actin (1:500) which cross-reacts with murine α-actin. This contractile protein was demonstrated in cells within the walls of the blood vessels. Some sections were stained with haematoxylin and eosin (H&E).

*Blood flow measurement*

To determine of local blood flow in control and tumour-bearing implants, the mice were anaesthetised with Hypnorm/Hypnovel as before and a 10 µl bolus of $^{133}$Xe, containing $10^3$ counts per s, was injected into the sponge implant via the cannula which was quickly plugged to prevent evaporation of the gas. The washout of the radioactive tracer was followed by external detection with a collimated gamma-scintillation detector (sodium iodide-thalium activated crystal; 1 inch by 1 inch) positioned 1 cm directly above the site of the injection. The radioactivity was accumulated for 40 s over 6 min after injection and the 40 s counts printed automatically on an SR7 scaler ratemeter (Nuclear Enterprises Ltd., London, UK). The rate of washout of $^{133}$Xe was expressed in terms of its half-time (t1/2; time taken for the radioactivity to fall to 50% of its original value).

*Assessment of pharmacological reactivity of newly formed blood vessels in control and tumour-bearing implants*

In any single implant, the untreated t1/2 value was measured following the injection of saline alone. Then 40 min later the t1/2 value was established following the administration of a specific vasoconstrictor. Up to three successive $^{133}$Xe washout assays, with 45 min intervals, were possible in one implant per day. At the end of the experimental session, the animal was kept warm until it had recovered fully from anaesthesia. Preliminary experiments showed that untreated t1/2 values were constant from day to day after day 8 (i.e. 6 days after cell injection) in the tumour-bearing implants and from day 10 for control implants; successive assays on the same day also gave constant t1/2 values.

Responses of the vessels to vasoconstrictors PAF (0.1, 0.5, 1 and 2 µg), endothelin-1 (0.125 and 1.25 ng), angiotensin II (0.05 and 0.5 µg), adrenalin (0.05, 0.5 and 5 µg) and 5-hydroxytryptamine (5-HT) (0.1 and 1 µg) or sterile saline were measured. Injections of the vasoactive agents were made into the sponges (50 µl) and skin (10 µl), immediately before the $^{133}$Xe solution was given.

*Chemicals*

Xenon injection, $^{133}$Xe (10 mCi in 3 ml) was obtained from Amersham International, UK. Platelet activating factor (PAF; 1-0- alkyl - 2 - acetyl - sn-glycero-3-phosphorycholine), angiotensin II, adrenalin, 5-hydroxytrytamine (5-HT) and monoclonal anti α-smoooth muscle actin clone 1A4 all came from Sigma Chemical Co. Ltd.; endothelin-1 porcine was from Peninsula Laboratories, Inc., (Belmont, Calif, USA); Hypnorm (0.315 mg ml$^{-1}$ fentanyl citrate and 10 mg ml$^{-1}$ fluanisone acetate) was purchased from Janssen Pharmaceuticals, Oxford, UK; and Hypnovel (5 mg ml$^{-1}$ of midazolam hydrochloride) was bought from Roche Pharmaceuticals, Welwyn Garden City, UK.

*Statistical analysis*

Results are given as mean(± s.e.m.) values from n animals. Comparison between groups was made with Student's *t*-test for unpaired data and a *P* value less than 0.05 was considered significant.

### Results

The angiogenic effect of tumour cells was assessed in the subcutaneous sponge implants by the progressive fall in $^{133}$Xe t1/2, i.e. progressive increase in local blood flow. In the control sponges, the $^{133}$Xe decreased from 26 ± 4 min at day 4 to reach t1/2 of normal skin values 5 ± 1 min by 14 days postimplantation. In the presence of tumour cells, this process was accelerated and normal skin values were attained before 10 days (i.e. 8 days after tumour cell injection) with a very marked effect at day 7 postimplantation (tumour t1/2 = 7 ± 1; control t1/2 = 15 ± 2 min). At this stage (7 days) in histological sections of the sponges, using the routine H&E staining, capillary-like structures were evident in tumour bearing implants but not in the control implants. However, at 14 days postimplantation blood vessels were evident in both types of implants. Histological examination of the sections stained for α-actin immunoreactivity showed positive reaction in control and tumour-bearing implants with no obvious difference between the two groups (Figure 1).

*Responses of the vasculature in control and tumour-bearing animals*

The effects of a number of vasoconstrictors were measured firstly in normal skin. These results (Table I) showed that the doses used caused marked increases in t1/2 values as a consequence of vasoconstriction.

The vasoconstrictors were then assessed for their effect on the vasculature in the implants. In the first set of such experiments, the vasomotor response to endothelin-1 was measured at day 10 postimplantation in both types of implant (i.e. 8 days after tumour cell injection in tumour-bearing implants). The initial washout rate of $^{133}$Xe in control implants (7.8 ± 1 min; $n = 4$) was not different from that in tumour-bearing implants (6 ± 1 min; $n = 4$) under these conditions. After a bolus injection of endothelin-1 (1.25 ng), blood flow in both implants decreased, i.e. t1/2 increased markedly, in both control and tumour-bearing implants (Figure 2). However, at the later stage of 14 days postimplantation, the t1/2 values in untreated implants of either type were again equal (5.1 ± 0.7 min, control; 6 ± 1.6 min, tumour-bearing implant; $n = 4$), and the response to endothelin-1 (1.25 ng) in control implants was marked by a substantial increase in t1/2 value (29 ± 3 min) but the tumour response was now substantially less than that seen at day 10, or that manifest by 14 day control sponge, with a t1/2 of 11 ± 1 min. (Figure 2).

Because of this divergence between control and tumour vessel response, all subsequent tests of vasoreactivity were made in implants at 14 days. Figure 3 shows the blood flow in control implants and the blood flow in tumour-bearing implants in response to four different vasoconstrictors. Over a range of doses for each vasoconstrictor there was a response as shown by the increase in t1/2 in the control implants. By contrast, the vessels in the tumour-bearing implants did not respond to PAF (0.1; 1 and 2 µg) or to low doses of endothelin-1, angiotensin II and adrenalin.

**Figure 1** Photomicrograph of control sponge implants **a**, (day 14 postimplantation) and Colon 26-bearing implants **b**, (day 14 postimplantation, i.e. 12 days after tumour cell injection) immunostained for α-smooth muscle actin (Streptavidin-peroxidase staining). Positive reaction is localised in the walls of vessels (arrows). Bars = 20 μm.

**Table I** Effects of local injected vasoconstrictors on the washout of $^{133}$Xe from skin

| | | *t1/2 (min) of $^{133}$Xe washout* |
|---|---|---|
| Untreated skin | (10) | 5 ± 1.8 |
| PAF | 0.1 μg (4) | 6 ± 1 |
| | 1 μg (7) | 12 ± 1[a] |
| Endothelin-1 | 0.125 ng (5) | 14 ± 2[a] |
| | 1.25 ng (8) | 22 ± 1[a] |
| Angiotensin II | 0.05 μg (4) | 10 ± 0.3[a] |
| | 0.5 μg (5) | 15 ± 1[a] |
| 5-HT | 0.1 μg (6) | 11 ± 1.5[a] |
| | 1 μg (7) | 15 ± 2[a] |
| Adrenalin | 0.05 μg (3) | 9 ± 1[a] |
| | 0.5 μg (3) | 19 ± 3[a] |

The values in the Table are the mean ± s.e.m. from the number of animals shown in parentheses. [a]$P < 0.05$, different from untreated skin values.

Significant responses were obtained at higher doses of the mediators but markedly less than in the control implants. Responses to 5-HT were generally similar in both control and tumour-bearing implants in that no significant differences in t1/2 values were recorded between the two groups at any of the doses used (Figure 3c).

### Discussion

Our results have confirmed the angiogenic effects of an adenocarcinoma cell line growing in subcutaneously implanted sponge discs (Mahadevan & Hart, 1991). These implants provide a well-defined, initially avascular compartment for the growth of tumour cells. Neovascularisation of the implant was assessed by $^{133}$Xe washout and has been correlated with histological evidence of vessel growth (Andrade *et al.*, 1987); the progressive fall in t1/2 reflected development of blood vessels in the implant. In the present experiments using this technique early changes in blood flow could be detected even before visible growth of the tumour mass was apparent.

The combination of sponge implant and $^{133}$Xe washout offers a valuable experimental opportunity to study the pro-

**Figure 2** Responses of Colon 26 blood vessels to endothelin-1 during tumour development. Response of vessels to endothelin-1 was measured by the rate of $^{133}$Xe washout at day 10 and 14 postimplantation in control **a**, and tumour-bearing implants **b**. The t1/2 values from untreated implants are demonstrated (empty columns). The vasoconstrictor effect of the agent did not change from day 10 to day 14 in the control implants but was lost in the tumour-bearing implants from day 10 to day 14. The values shown represent mean ± s.e.m. from 4 animals per group. $^{*}P<0.05$; $^{**}P<0.01$, different from control.

**Figure 3** Vasoconstrictor effects of angiotensin II, adrenalin, 5-HT and PAF in tumour-bearing implants (●) and control implants (○) measured at day 14 after implantation. The values to the left of the break represent $^{133}$Xe washout from untreated implants. Although the untreated t1/2 values were identical for control and tumour-bearing implants, the vasoconstrictor effects (increased t1/2) were consistently lower (angiotensin II, endothelin-1) or absent (PAF) in tumour-bearing implants. Note that with 5-HT, both sets of implants showed identical responses. The values shown represent mean ± s.e.m. from 4–8 animals at each dose. $^{*}P<0.05$, $^{**}P<0.01$, different from control.

perties of newly formed blood vessels and to compare the response of vessels in neoplastic tissue with vessels in granulation tissue. Abnormal angioarchitecture has been demonstrated in granulation tissue; such abnormalities are comparable to those found in capillary sprouts in tumours (Warren, 1979). We have also observed that, in the absence of tumour cells, newly formed blood vessels infiltrating the sponge matrix exhibited features such as dilatation, tortuosity and saccular structure (Andrade unpublished results). All these features are apparent in tumour blood vessels (Jain, 1988; Vaupel *et al*., 1989).

The method allows non-destructive repeated measurements of blood flow in the same animal over the period of neovascularisation of the implants; thus changes occurring during the development of normal or tumour vasculature may be followed in the same animals. Consequently we were able to demonstrate clearly that there was a change in vaso-reactivity to endothelin-1 between 10 and 14 days in the tumour-bearing implants. A similar progressive loss in the capacity to react to vasoactive agents by blood vessels in experimental mammary tumours as the tumours enlarged was reported by Wickersham *et al*. (1977). A possible explanation for the progressive loss of reactivity in tumour, but not control, implants could be that, unlike the vessels of inflammatory granulation tissue, tumour vessels are characterised by a steady progression to necrosis without any intervening stable maturation phase (Suzuki *et al*., 1984). Another contributing factor could be that during neoplastic growth some of the preexisting host vessels incorporated in the tumour mass disintegrate, are obstructed or are compressed (Vaupel *et al*., 1989).

At the 14 day time point decreased sensitivity in the tumour neovasculature relative to the age-matched control neovasculature was the predominant response to most of the vasoconstrictors used in our experiments. However one vasoconstrictor, 5-HT, elicited very similar responses in all of the three vascular beds examined (normal skin, control implants and tumour implants). A normal vasoconstrictor response to 5-HT also has been reported in subcutaneously implanted Meth-A tumours (Stucker *et al*., 1991). Several studies have suggested that arteries and arterioles are resistant to neoplastic invasion (Intaglietta *et al*., 1977; Warren, 1979). Possibly intact innervation and contractile elements may remain in normal blood vessels incorporated in the invading tumour mass, thus maintaining the ability to respond to serotoninergic agonists. A weak response was obtained to angiotensin II and adrenalin in neoplastic tissue only after administration of a dose 10–100 fold greater than that which evoked a response in the control neovasculature. Suzuki *et al*. (1984) observed that newly growing tumour vessels did not react to topically administered angiotensin II. The vessels supplying tumours have been reported to be relatively unreactive to locally applied drugs which act on smooth muscle (Hirst *et al*., 1991). This lack of response might reflect a lack of structural strength, with tumour vessels having little smooth muscle and a poorly organised adventitia (Can *et al*., 1984). Interestingly then our immunohistochemical studies failed to show any marked differences in actin content between control and tumour-bearing implants (Figure 1). Though this assessment is, of necessity, crude it could suggest that decreased sensitivity in Colon 26 tissue is not due to a lack of contractile elements in tumour blood vessels. Perhaps other factors could contribute to the differences observed in our system. For example, tumour vascular endothelium possesses immature cell contacts within the endothelial lining of the vessel wall (Warren, 1979; Jain, 1988). We have shown that the pharmacological responses of blood vessels in a growing tumour, Colon 26, differed considerably from the responses of vessels of a similar age which were not associated with neoplastic tissue. We have observed similar pharmacological differences between normal and B16 melanoma neovasculature (unpublished observations) suggesting this response is not a unique property of the Colon 26 tumours. These findings suggest that the nature of the angiogenic stimulus influences the pharmacological behaviour of newly formed blood vessels. The experimental system described here may be of some utility in the search for therapeutically useful, pharmacological differences between normal and tumour vasculature.

We thank the Imperial Cancer Research Fund for funding S.P.A.

### References

ANDRADE, S.P., BAKHLE, Y.S. & PIPER, P.J. (1991). Decreased responses to platelet activating factor (PAF), endothelin-1 (ET-1) and angiotensin II (AII) in tumor blood vessels in mice. *Br. J. Pharmacol.*, **104**, 422p.

ANDRADE, S.P., FAN, T.-P.D. & LEWIS, G.P. (1987). Quantitative *in vivo* studies on angiogenesis in a rat sponge model. *Br. J. Exp. Path.*, **68**, 755–765.

CHAN, C.R., BABBS, C.F., VETTER, J.R. & LAMAR, C.H. (1984). Abnormal response of tumor vasculature to vasoactive drugs. *J. Natl Can. Inst.*, **72**, 145–150.

FOLKMAN, J. & KLAGSBRUN, M. (1987). Angiogenic factors. *Science*, **245**, 442-447.

HIRST, D.G., HIRST, V.K., SHAFFI, K.M., PRISE, V.E. & JOINER, B. (1991). The influence of vasoactive agents on the perfusion of tumors growing in three sites of the mouse. *Int. J. Radiat. Biol.*, **60**, 1/2, 211–218.

INTAGLIETTA, M., MYERS, R.R., GROSS, J.F. & REINHOLD, H.S. (1977). Dynamics of microvascular flow in implanted mouse mammary tumors. *Bibliot. Anat.*, **15**, 273–276.

JAIN, R.K. (1988). Determinants of tumor blood flow: A Review. *Cancer Res.*, **48**, 2641–2658.

KALLMAN, R.F., DENARDO, G.L. & STASH, M.J. (1972). Blood flow in irradiated mouse sarcoma as determined by the clearance of Xenon-133. *Cancer Res.*, **32**, 483–490.

MAHADEVAN, V. & HART, I.R. (1991). Divergent effects of flavone acetic acid on established versus developing tumor blood flow. *Br. J. Cancer*, **63**, 889–892.

MATTSSON, J., ALPSTEN, M., APPELGREN, L. & PETERSON, H.-I. (1980). Influence of noradrenaline on local tumor blood flow. *Eur. J. Cancer*, **16**, 99–102.

MATTSSON, J., LILJA, J. & PETERSON, H.-I. (1982). Influence of vasoactive drugs on local tumor blood flow. *Eur. J. Cancer*, **18**, 677–684.

PETERSON, H.-I. (1979). Tumor blood flow compared with normal tissue blood flow. In *Tumor blood flow circulation: angiogenesis, vascular morphology and blood flow of experimental and human tumors*. Peterson, H.-I. (ed.) pp. 103–114 CRC Press: Florida.

SCHOEFL, G.I. (1963). Studies on inflammation III. Growing capillaries: Their structure and permeability. *Virch. Arch. Path. Anat.*, **337**, 97–141.

STUCKER, O., VICAUT, E. & TEISSEIRE (1991). Hyper-responsiveness to 5-HT agonists by tumor-linked arterioles in mice: consequences for tumor growth. *Int. J. Radiat. Biol.*, **60**, 237–241.

SUZUKI, M., HORI, K., ABE, I., SAITO, S. & SATO, H. (1984). Functional characterization of the microcirculation in tumor. *Cancer Metast. Rev.*, **3**, 115–126.

THIEDE, K., MOMBURG, U., ZANGEMEISTER, P. SCHLAG & SCHIRRMACHER (1988). Growth and metastasis of human tumors in nude mice following tumor-cell inoculation into a vascularized polyurethane sponge matrix. *Int. J. Cancer*, **42**, 939–945.

TSURO, T., YAMORY, T., NAGANUMA, K., TSUKAGOSHI, I. & SAKURAI, Y. (1983). Characterisation of metastatic clones derived from a metastatic variant of mouse colon adenocarcinoma. *Cancer Res.*, **43**, 5437–5442.

TVEIT, E., LUNDSTAM, S., HULTBORN, R. & WEISS, L. (1981). Haemodynamics of human renal adenocarcinoma. *Bibliot. Anat.*, **20**, 624–627.

VAUPEL, P., KALLINOWSKI, F. & OKNIEFF, P. (1989). Blood flow, Oxygen and Nutrient Supply and Metabolic Microenvironment of Human Tumors: A Review. *Cancer Res.*, **49**, 6449–6459.

WARREN, B.A. (1979). Tumor angiogenesis. In *Tumor Blood Circulation: angiogenesis, vascular morphology and blood flow of experimental and human tumors*. Peterson, H.-I. (ed.) pp. 49–75, CRC Press: Florida.

WICKERSHAM, J.K., BARRET, W.P., FURUKAWA, S.B., PUFFER, W.H. & WARNER, N.E. (1977). An evaluation of the response of the microvasculature in tumors in C3H mice to vasoactive drugs. *Bibliot. Anat.*, **15**, 291–293.

YOUNG, S.W., HOLLENBERG, N.K., KAZAM, E., BERKOWITZ, D.M., HAINEN, R., SANDON, T. & ABRAMS, H.I. (1979). Resting host and tumor perfusion as determinants of tumor vascular responses to norepinephrine. *Cancer Res.*, **39**, 1898–1905.